# The level of knowledge and associated socio-demographic factors on cervical cancer among women: a cross-sectional study at Kenyase Bosore community, Ghana

**DOI:** 10.11604/pamj.2019.34.44.19471

**Published:** 2019-09-24

**Authors:** Ampofo Ama Gyamfua, Isaac Nkrumah, Bukola Mary Ibitoye, Beatrice Ampofo Agyemang, Evelyn Serwaa Ofosu, Joyce Mahlako Tsoka-Gwegweni, Samuel Nambile Cumber

**Affiliations:** 1 Garden City University College, Kumasi, Ghana; 2 Kwame Nkrumah University of Science and Technology Hospital, Kumasi, Ghana; 3 Department of Nursing Science, University of Ilorin, Ilorine, Nigeria; 4 Faculty of Health Sciences, University of the Free State, Bloemfontein, South Africa; 5 School of Health Systems and Public Health, Faculty of Health Sciences, University of Pretoria Private Bag X323, Gezina, Pretoria, 0001, Pretoria, South Africa; 6 Section for Epidemiology and Social Medicine, Department of Public Health, Institute of Medicine (EPSO), The Sahlgrenska Academy at University of Gothenburg, Box 414, SE-405 Gothenburg, Sweden

**Keywords:** Knowledge, socio-demographic factors, cervical cancer, Ghana

## Abstract

**Introduction:**

Cervical cancer is a major cause of death amongst women around the world. In Ghana, it accounts for over 2,119 female deaths and about 3,151 new diagnoses of the disease. It is usually diagnosed at an advanced stage, making it difficult to treat. This study aims at assessing the knowledge on risk factors, prevention and treatment of cervical cancer among women in Kenyase Bosore, Ghana.

**Methods:**

This study was a cross-sectional descriptive study conducted among women in Bosore Kenyase, Ghana. A total of 200 women were selected for the studies using the convenience sampling technique. Structured questionnaires were used for data collection and statistical package for social sciences application was also used to analyse the data. Pearson chi-square test was used to find associations between knowledge and awareness level and socio-demographic characteristics of the participants.

**Results:**

Overall, 9.7% of the respondents had high knowledge on cervical cancer, 20.6% had moderate knowledge and 69.7% had low knowledge on cervical cancer. There was a significant association between educational background (p=0.000) and awareness level of the respondents. There was also a significant association between the occupation (p=0.003), educational background (p=0.000) and knowledge level of the respondents.

**Conclusion:**

The knowledge level of the respondents was very low. Specifically, the respondents had inadequate knowledge on risk factors, signs and symptoms, prevention and treatment of cervical cancer. The authors recommend the intensification of cervical cancer education in Kenyase Bosore, and Ghana as a whole.

## Introduction

Cervical cancer is a malignant cancer of the cervix that begins as a slight abnormal squamous cellular change, or dysplasia [1]. If left untreated, these cells may progress into severe dysplasia, also known as High-Grade Squamous Intraepithelial Lesions (HSIL), and then onto invasive carcinoma. Detecting cervical cancer while it is early invasive or pre-invasive significantly improves the probability of curing the disease [2]. Once the cancer has spread to the lymphatic system or parametrical tissue adjacent to the cervix, successful treatment is less likely. Cervical cancer most often in women older than 40 years, but can occur in younger women as well [3]. The risk for cancer of the cervix depends on a woman's sexual history, immune system, health status, and lifestyle [4]. The risk factors include early sexual debut, having multiple sexual partners or having sex with someone who has multiple sexual partners, having sex at an early age (younger than 18 years because they increase the chance of getting human papillomavirus (HPV). Women who have problems with their immune system are at increased risk of cervical cancer, especially if they have been exposed to HPV [5]. Factors that affect the immune system and can increase the risk of cancer of the cervix are smoking, Human Immunodeficiency Virus (HIV) infection, history of sexually transmitted infections (STIs), a family history of cervical cancer, older age, and poverty [5]. Other risk factors include. History of high-grade squamous intraepithelial lesions, history of cancer of the cervix, vagina, or vulva, and history of not getting routine Pap tests [5]. A Papanicolaou smear is a screening procedure that provides secondary prevention in the development of cervical cancer by detecting dysplastic cellular changes that may lead to cervical abnormalities [6]. The Pap smear has been the preferred prevention strategy for cervical cancer by periodically screening women to identify abnormal cells, to determine the need for additional procedures that can destroy the abnormal cells before they progress into invasive cancer [7].

Approximately 90% of deaths from cervical cancer occurr in low and middle-income countries [8]. In Ghana, current estimates indicate that every year 3151 women are diagnosed with cervical cancer and 2119 die from the disease [9]. Cervical cancer incidence peaked in women 75 to 79 years of age in the Ashanti region and 70 to 74 years old in the Greater Accra region [10]. It is possible that exposure to HPV may have been, on average, at a younger age in the Greater Accra region [10]. Thus screening should be performed at least once for every woman in the target age group (30-49 years) when it is most beneficial [11]. The World Health Organization (WHO) has estimated additional cases of cervical cancer to be over 5,000 in Ghana with at least 3,300 deaths every year by 2025. The high incidence of cervical cancer could be attributed to lack of knowledge on cervical cancer which usually results in late diagnosis at an advanced stage making it very difficult to treat [12]. The high mortality rate and its projected increase could be reduced through a comprehensive approach that includes prevention, early diagnosis and effective screening and treatment programs. Thus greater proportions of women of reproductive age are at high risk of developing cervical cancer as there is no systematic national screening or treatment available [13,14]. In the absence of this, most of the cervical cancer screening that takes place in the country can be described as opportunistic screening, where doctors request Pap smears or VIA for patients who are seen in clinics for either general medical examinations or for consultations unrelated to cervical cancer. Also, various Non-governmental organizations organize a free screening for cervical cancer at various communities, however, most have recorded low rates of patronage of cervical cancer screening services [10,13,15]. This has therefore, necessitated the need for the study to assess the knowledge level on risk factors, signs and symptoms, prevention, treatment of cervical cancer among women in Kenyase Bosore community in Ghana.

## Methods

A descriptive and analytic cross-sectional study design was carried out among the female population of the inhabitants of Bosore community, Ashanti Region, Ghana who were of the ages of fifteen [15] years and above. The study was conducted in Bosore in the Ashanti Region. Bosore is a town in the Kwabre North District in the Ashanti Region of Ghana. It is about 10 kilometres from Kumasi, the regional capital. The total population of the study area is about 9,400 inhabitants. The main ethnic composition of the study area is Akan people mostly Ashanti followed by northerners and other ethnic backgrounds. The predominant occupation in the area is petty trading. The literacy rate in the town is average with parents having their children in educational institutions. Inhabitants of the community seek healthcare from healthcare and few private hospitals. All women who were residents of the community and willing to participate in the study were included. However, women below age 18 were excluded. A convenience sampling technique was used to select the respondents from their shops and homes, however, respondents were chosen without any preferences. Using the Cochran formula, a sample size of 200 was calculated and used for the study. A well-structured questionnaire which was developed based on the review of related journals was used. The pre-test or pilot study was conducted among 10 women to ascertain the contents and clarity of the questionnaire. The cronbach's alpha which is a measure of internal consistency ranging from 0.70 to 1.00, with higher coefficients indicating higher levels of reliability was used to determine the validity and the reliability of the questionnaire.

Cronbach's alpha of most of the questions was 0.934. Changes were made to modify the questionnaire after the pilot study and the entire questionnaire was available in English. The questionnaire was then personally delivered to the women in paper form by the researchers. In situations where participants were illiterates, the researchers explained the questions to them in the local dialect. The questionnaire consisted of 25 open and close ended questions with three main parts. The first part contained a series of socio-demographic questions (e.g. age, marital status, educational level, occupation). The second part included items about common cervical cancer knowledge such as risk factors and symptoms and also about cervical cancer screening knowledge. Each section contained questions on the awareness, signs and symptoms, risk factors, prevention and treatment. Each section was scored over 20 and divided by the number of the items to obtain the scores. The total score ranging from 65-100% was defined as high level of knowledge, scores ranging from 40-64% was defined as fair or moderate knowledge level of knowledge and scores 1-39% were classified as low knowledge level. Data were analyzed using the Statistical Package for Social Sciences (SPSS) application 21.0 and presented in tables. Categorical variables were summarized as frequencies and percentages, and Pearson chi-square test was used to test associations. The level of significance was set at a p-value of 0.05.

**Ethical considerations:** an introductory letter was sought from Garden City University College and was given to the Unit Committee leader of the community. The research proposal was reviewed and approval was given by the Unit committee leader to proceed with the study. The consent of respondents was sought before data collection was started. Respondents' freedom to partake or withdraw from the study at any time was respected and confidentiality and anonymity of respondents were guaranteed.

## Results

**Demographic characteristics:** two hundred (200) women were interviewed. More than half of the respondents (143, 71.5%) were between 15-40 years. Most of the respondents (92, 46%) were married. The majority (59) 29.5% were illiterate. Most (144, 72%) of the respondents were Christians. Most of the respondents were predominantly traders representing 45.5%. More than half (52%) of the respondents had their first menarche between the ages of 12- 14 years. Most of the respondents (45%) had their first birth between the ages of 19-25 years, Almost all (96%) of the respondents were not smokers. Whilst 54.5% of the respondents had between 1-5 children (Table 1).

**Table 1:** Socio-demographic characteristics of women in Kenyase Bosore, community, Ghana

VARIABLES	FREQUENCY N=200	PERCENTAGE (%)
**AGE**		
Above 4O	57	28.5
15-40	143	71.5
**MARITAL STATUS**		
Divorce	28	14.0
Married	92	46.0
Single	68	34.0
Widow	12	6.0
**RELIGION**		
Christian	144	72.0
Muslim	55	27.5
Traditional	1	.5
**EDUCATIONAL BACKGROUND**		
Illiterate	59	29.5
Primary	28	14.0
JHS	54	27.0
SHS	33	16.5
Tertiary	26	13.0
**OCCUPATION**		
Teacher	10	5.0
Traders	91	45.5
Farmers	14	7.0
Students	28	14.0
Unemployed	25	12.5
Hairdresser	13	6.5
Nurse	6	3.0
Seamstress	13	6.5
**AGE AT FIRST MENARCHE**		
15-16	96	48.0
12-14	104	52.0
**AGE AT FIRST BIRTH**		
33 ABOVE	4	2.0
26-32	43	21.5
19-25	89	44.5
NONE	64	32.0

**Awareness of cervical cancer:** a majority (111, 55.5%) of the respondent had heard of cervical cancer. 33% of the respondents said they heard of cervical cancer from the media, followed by 43% had never heard of it, 29, 14.5% of the respondents reported that they had heard about cervical cancer from some health personnel, 5.5% heard about it from the church, 3.5% reported they heard of it from their teachers, 1.5% heard about cervical cancer from friends. The majority (130, 65%) of the respondent said cervical cancer can be treated at the early stage. 58% of the respondents don't know if a healthy-looking person can have cervical cancer. 47% of the women surveyed said that cervical cancer is curable when diagnosed early (Table 2).

**Table 2:** Awareness on cervical cancer among women in Kenyase Bosore, community, Ghana

VARIABLE	FREQUENCY (n)	PERCENTAGE (%)
Have you heard of Cervical cancer?		
Yes	111	55.5
No	89	42.5
If Yes, source of information		
Media	64	32
Health personnel	29	14.5
Teachers	7	3.5
Friends	3	1.5
Church	11	5.5
None	86	43
Can cervical cancer be treated at early stage?		
Yes	130	65
No	70	35
Can healthy looking person have cervical cancer?		
Yes	60	30
No	140	70
Is cervical cancer curable when diagnosed early?		
Yes	94	47
No	108	53
Is cervical cancer preventable through vaccination?		
Yes	33	16.5
No	167	83.5
Is cervical cancer preventable through screening?		
Yes	33	16.7
No	167	83.5

**Association between socio-demographic factors and awareness on cervical cancer:** there was a significant association between educational status and awareness of cervical cancer (p=0.000). There was no significant association between awareness and age, occupation, religion, marital status and age at first parity (Table 3).

**Table 3:** Association between socio-demographic factors and awareness of cervical cancer among women in Kenyase Bosore, community, Ghana

	AWARENESS OF CERVICAL CANCER			X^2^ VALUES	SIG. VALUES
	YES	NO	TOTAL		
**AGE**					
15-40	79(55.2%)	64(44.8%)	143		
40 above	32(56.1%)	25(43.9%)	57	1.260	0.739
**OCCUPATION**					
Teachers	10(100%)	0(0%)	10		
Traders	44(48.3%)	47(51.7%)	91		
Nurse	5(83.3%)	1(16.7%)	6		
Hairdressers	5(38.5%)	8(61.5%)	13		
Students	18(64.3%)	10(35.8%)	28		
Farmers	10(71.4%)	4(21.6%)	14		
Unemployed	14(56.0%)	11(44.0%)	25		
Seamstress	5(38.5%)	8(61.5%)	13	23.349	0.326
**MARITAL STATUS**					
Single	42(61.8%)	26(38.8%)	68		
Married	43(46.7%)	49(53.3%)	92		
Divorced	19(67.9%)	9(45.5%)	28		
Widow	6(54.5%)	5(45.5%)	11	17.143	0.114
**EDUCATION**					
SHS	25(75.8%)	8(24.2%)	33		
JHS	31(57.4%)	23(42.6%)	54		
Illiterate	18(30.5%)	41(69.5%)	59		
Primary	13(46.4%)	15(53.6%)	28		
Tertiary	24(92.3%)	2(7.7%)	26	42.250	0.000
**RELIGION**					
Christianity	81(56.2%)	63(43.8%)	144		
Muslim	29(52.7%)	26(47.3%)	55		
Traditional	0(100.0)	0(100.0)	0	2.299	0.890
**AGE AT FIRST BIRTH**					
19-25	43(48.3%)	46(51.6%)	89		
26-32	27(62.8%)	16(37.2%)	43		
33above	2(50.0%)	2(50.0%)	4	4.219	0.837

P value <0.05 = significant

**Respondents' knowledge level on cervical cancer:** overall, most of the respondents had low knowledge about cancer of the cervix which majority (69.7%) scoring between 1-39% and 9.7% scoring between 100-65%). In detail, knowledge on the signs and symptoms, risk factors, its prevention, treatment of cervical cancer among others was very low (Table 4). Respondents who had high knowledge of cervical cancer were educated whilst respondents who were illiterate had low knowledge. Also, respondents who had high knowledge on cervical cancer were nurses and those who had low knowledge of cervical cancer were mostly traders. It was also revealed that the respondents who fell between the ages of 15-40 years had moderate or fair knowledge while those who are above 40 years had low knowledge on cervical cancer. As indicated on Table 4, the study also revealed that married women had moderate knowledge of cervical cancer.

**Table 4:** Respondents' knowledge level on cervical cancer among women in Kenyase Bosore, community, Ghana

Questions	High (Number of respondents who scored between100-65%)	Moderate (Number of respondents who scored between64-40%)	Low (Number of respondents who scored between39-1%)
Knowledge on signs and symptoms of cervical cancer	6(3%)	45(22.5%)	149(74.5%)
Knowledge on risk factors of cervical cancer	18(9%)	30(15%)	152(76%)
Knowledge on prevention of cervical cancer	21(10.5%)	43(21.5%)	136(68%)
Knowledge on treatment of cervical cancer	18(9%)	53(26.5%)	129(64.5%)
Overall knowledge on cervical cancer (%)	9.7%	20.6%	69.7%

**Knowledge on sign and symptoms of cervical cancer:** majority of the respondents identified that they had no knowledge of the following being signs and symptoms of cervical cancer vaginal bleeding between periods (150, 75%), persistent vaginal discharge with unpleasant smell (160, 80%), discomfort or pain during sex (191, 93.6%), vaginal bleeding after menopause (192, 94%) and vaginal bleeding during/after sex (191, 93.6%) as possible warning signs and symptoms of cervical cancer. On another hand, the respondents were affirmative that having heavier menstrual period than usual was one of the signs and symptoms of cervical cancer.

**Knowledge on risk factors of cervical cancer:** the respondents had no knowledge about long term use of oral contraceptives, infections with HPV, have weakened immune systems, not going for pap smear test among others showing (144, 86%), (144, 72%), (140, 70%), (165, 82.5%) respectively as the risk factors to cervical cancer. However, 52% of the respondents also indicated that having many sex partners can be a risk factor for cervical cancer. However, 52% of the respondents also indicated that having many sex partners can be a risk factor to cervical cancer. In conclusion, it can be argued that most of the respondents don't know about the risk factors that likely to cause cancer of the cervix.

**Knowledge on prevention of cervical cancer:** sexually transmitted diseases as a means of preventing cancer of the cervix was well indicated by most (112, 56%) of the respondents. However, it was further indicated that most of the respondents had no knowledge about the other preventive measures: for example, vaccination against cervical cancer and cervical cancer screening programmes representing (165, 82.5%) and (150, 75%) respectively. In conclusion, most of the respondent had no knowledge about some of the preventive measures that could be applied to cancer of the cervix.

**Knowledge of treatment of cervical cancer:** sixty six percent of the respondents were of the view that cervical cancer can be treated through surgical means whereas 34% said no to the statement. However, (147, 73.5%) and (181, 90.5) of the respondents had no knowledge about early treatment of cervical cancer before it spreads to the other organs of the body and radiotherapy respectively. It can be concluded that respondents only had knowledge about surgical treatments of cervical cancer (Table 5).

**Table 5:** Knowledge on risk factors, signs and symptoms, prevention and treatment of cervical cancer among women in Kenyase Bosore, community, Ghana

RISK FACTORS	Yes n(%)	No n(%)
Infection with HPV	56(28)	144(72)
Excessive alcohol intake	11(5.5)	189(94.5 )
Having a weakened immune system	60(30)	140(70)
Long term use of the oral contraceptive pill	28(14)	172(86)
Having many sexual partners	104(52)	96(48)
Not going for regular smear test(Pap)	35(17.5)	165(82.5)
**SIGNS AND SYMPTOMS OF CERVICAL CANCER**		
Bleeding between Periods	50(25%)	150(75)
Unusual vaginal discharge	40(20)	160(80)
Severe lower abdominal pain	47(21.5)	153(79.5)
Vaginal bleeding during/after sex	4(6.4)	191(93.6)
**PREVENTION OF CERVICAL CANCER**		
Cervical cancer screening programs	50(25)	150(75)
Vaccination against cervical cancer	35(17.5)	165(82.5)
Treating sexually transmitted infections	112(56)	88(44)
Avoidance of smoking	58(29)	142(71)
**TREATMENT OF CERVICAL CANCER**		
Treat before it spreads	53(26.5)	147(73.5)
Surgical means	132(66)	68(34)
Radiotherapy	19(9.5)	181(90.5)

**Association between the socio-demographic factors and the knowledge level of cervical cancer:** there was a significant association between occupation and educational status with a p-value of (0.003 and 0.000) respectively. As shown in Table 6, the remaining factors were not statistically significant.

**Table 6:** Association between the socio-demographic factors and the knowledge level among women in Kenyase Bosore, community, Ghana

SOCIODEMOGRAPHIC FACTORS	KNOWLEDGE LEVEL OF CERVICAL CANCER				
	HIGH(65-100)	MODERATE(40-64)	LOW (1-39)	X^2^ VALUES	SIG. VALUES
**AGE**					
15-40	41(28.7%)	9(6.3%)	93(65%)		
40 above	15(26.3%)	3(5.3%)	39(68.4%)	0.224	0.894
**OCCUPATION**					
Teacher	4(40%)	1(10%)	5(50%)		
Traders	24(26.4%)	5(55.%)	62(68.1%)		
Nurse	5(83.3%)	1(16.7%)	0(0%)		
Hairdressers	2(15.4%)	0(0%)	11(84.6%)		
Students	8(28.6%)	0(0%)	20(71.4%)		
Farmer	7(50%)	3(21.7%)	4(28.6%)		
Unemployed	5(20%)	2(8%)	18(72%)		
Seamstress	1(7.7%)	0(0%)	12(92.3%)	32.903	0.003
**MARITAL STATUS**					
Single	17(25%)	7(10.3%)	44(64.7%)		
Married	28(30.4%)	3(3.3%)	61(66.3%)		
Divorced	6(21.4%)	0(0%)	22(78.6%)		
Widow	4(36.4%)	2(18.2%)	5(45.5%)	12.725	0.122
**EDUCATION**					
SHS	9(27.3%)	0(0%)	24(72.7%)		
JHS	20(37%)	4(7.4%)	30(55.6%)		
Illiterate	6(10.2%)	4(6.8%)	49(83.1)		
Primary	4(14.3%)	2(7.1%)	22(78.6%)		
Tertiary	17(65.4%)	2(7.7%)	7(26.9%)	35.946	0.000
**RELIGION**					
Christianity Muslim	39(27.1%) 16(29.1%)	7(4.9%) 5(9.1%)	98(68.1%) 34(61.8%)		
Traditional	1(100%)	0	0	4.063	0.398
**AGE AT FIRST BIRTH**					
19-25	26(29.2%)	5(5.6%)	58(65.2%)		
26-32	13(30.2%)	2(4.7%)	28(65.1%)		
33 above	1(25%)	0(%)	3(75%)	1.162	0.979

## Discussion

**Knowledge and awareness level of cervical cancer:** cervical cancer is the leading cause of death from gynaecological cancers in Ghana, yet knowledge about its causes among the general population and health care providers is limited. Women with knowledge on cervical cancer respond positively to the great need for screening (Muri, 2006). The present study showed that the level of awareness on cervical was low (55.5%), Aswathy *et al.* in a study in rural Kerala also reported that 72.1% of the women were aware of cervical cancer [16]. Thus the current finding is much lower than the level of awareness reported in Aswathy *et al.* Also that current finding is similar to the work done by [17] which was carried out on college girls in India that explored only knowledge levels, reported low levels of awareness (20%). WHO (2019) has reported that knowledge and awareness of cervical cancer in other developing countries have reported low awareness of the disease [11]. Thus Ghana being a developing country is no exception which the present the study has alluded to. This may be attributed to the fact that the study population were dominated mostly illiterate and traders (45.5%). The overall knowledge on cervical cancer was low with most of the respondents (69.7%) scoring less than 40%, however, tertiary students scored high to moderate scores (40-100%) with majority scoring between 69-100%. This is in contrast with [17] who reported a low level of knowledge on cervical cancer among the graduate and postgraduate students of some leading women's colleges of Kolkata, India. Also, it was revealed from the study that the respondents (76%) had no knowledge about the risk factors of cervical cancer, which increases a woman's chances of getting cervical cancer. However, most of the respondents (52%) were able to link sexual activity to an individual's risk of developing cervical cancer. This is similar to a study conducted in India which 41% students were aware of a link between sexual activity and cervical cancer likewise 38.4% was demonstrated by college students aged 18 to 35 years in Accra, Ghana [18]. In addition to this, a study conducted by [19] also mentioned that 45% of the respondents indicated multiple sexual partners and other promiscuous behaviors as the most common risk factor of cervical cancer. Similarly, in a Malaysian study, women aged 21-56 years could not identify any of these risk factors [20]. The present study appears to suggest that between 2009 and 2019 knowledge on risk factors of cervical cancer has improved in Ghana. Thus there is the need to enhance education on cervical cancer since the chances of getting cancer of the cervix could be very high owing to the inadequate knowledge of the risk factors. In addition to the above, all respondents could not recognize the signs and symptoms, prevention and treatment, however 56% of them were able to link the treatment of sexually transmitted infections to the preventive measure of cervical cancer. Only few (25%) of them were aware of screening and vaccination as a preventive measure. Nonetheless, majority of the respondents understand that treatment should be sought early if detected. This is line with studies that reported low awareness on screening and vaccination of cervical cancer [17,18,20].

**Association between socio-demographic and awareness level:** there was an association between educational background (p<0.000) and awareness level of the respondents in Bosore community. Most of the literates had high awareness while the majority of the illiterates were unaware of cervical cancer. This in line with a study by [21] in Tanzania who found a significant relationship (p<0.001) between educational status and awareness on cervical cancer. A study by [22] in London also confirmed a significant association between the socio-demographic factors and awareness level of respondents. Additionally, a study among a group of low-income uninsured women showed a significant association between the socio-demographic factors and awareness level [23].

**Association between socio-demographic and knowledge level:** the present study indicated a significant association between the occupation (p<0.003), educational background (p>0.000) and knowledge level of the respondents in Bosore community. The respondents having low to medium level of education scoring below 40% and almost all the tertiary students (65.4%) scoring above 65% showing a high knowledge level. This appears to suggest that individuals with tertiary education are more likely to have sufficient knowledge about cervical cancer. This phenomenon was observed by [21,24,25] amongst Ethiopians, Tanzanians, India respectively. It can therefore be speculated that the more literate there are in a population, the more knowledgeable they are likely to be with regards to cervical cancer. Thus developed countries with high literacy level are likely to have its individuals show high knowledge of cervical cancer.

## Conclusion

The study revealed that the knowledge level of the respondents was very low. In detail, the respondents have inadequate knowledge about risk factors of cervical cancer, which, increases a woman's chance of getting cancer of the cervix. Moreover, the study also revealed that the respondents' knowledge of the signs and symptoms of cervical cancer were very low. Again, respondents could not identify most of the prevention and treatment of cervical cancer. Furthermore, educational level and occupation of the individuals are likely to influence their knowledge of cervical cancer. Our recommendations include the introduction of educational programs to enhance knowledge of cervical cancer on media platforms such as television, radio programs among others in different languages. In addition to this, the distribution of educational materials on cervical cancer in various languages should be done to improve knowledge of cervical cancer. Finally, cervical cancer education at antenatal and family planning clinics in the community should also be encouraged.

**Limitations of the study:** the limitations of the study was mainly the language barrier this was because the researchers found it very difficult expressing some of the medical jargons in the local dialect. Thus may affect the responses given by the respondents. Therefore, interpretation of the results should be done carefully.

### What is known about this topic

- Cervical cancer is the second leading causes of cancer amongst women in Ghana and the world at large;
- Cervical cancer the leading cause of cancer deaths in Ghana;
- Knowledge and awareness of cervical cancer are very high in developing countries resulting in low mortality compared to developing countries.

### What this study adds

- Low awareness was reported amongst women in Kenyase, Bosore in Ghana;
- Majority of the women could not recognize the signs and symptoms of cervical cancer;
- High literacy level in developing countries may improve knowledge and awareness of cervical cancer in addition to this, health education on cervical cancer needs to be intensified.

## Competing interests

The authors declare no competing interests.
